# Diet in Knee Osteoarthritis—Myths and Facts

**DOI:** 10.3390/nu17111872

**Published:** 2025-05-30

**Authors:** Natalia Kasprzyk, Shreya Nandy, Bogna Grygiel-Górniak

**Affiliations:** 1 Department of Rheumatology, Rehabilitation and Internal Diseases, Poznan University of Medical Sciences, 61-701 Poznan, Poland; 2 Rheumatology Research Group, Department of Rheumatology, Rehabilitation and Internal Diseases, Poznan University of Medical Sciences, 61-701 Poznan, Poland

**Keywords:** osteoarthritis, aging, nutrition, macronutrients, micronutrients, symptomatic slow-acting drugs

## Abstract

Knee osteoarthritis (OA) is a common degenerative joint disease affecting global health. Its increasing prevalence, particularly among aging populations, remains a leading cause of disability. Besides conventional pharmacological and surgical treatments, dietary interventions are promising strategies to alleviate OA symptoms and progression. Unfortunately, scientific evidence does not support many commonly used, misleading ideas about nutrition in knee OA. Recent data highlight the detrimental effects of high-carbohydrate and high-fat diets, particularly those rich in refined sugars and saturated fats, which exacerbate systemic inflammation and contribute to cartilage degradation. Conversely, diets rich in omega-3 fatty acids, polyphenols, and dietary fiber have shown anti-inflammatory and chondroprotective properties. A Mediterranean diet rich in these nutrients effectively prevents the development of OA and its comorbidities, including obesity and cardiovascular disease. The role of supplements, such as glucosamine, chondroitin, and vitamin D, is questioned due to the lack of evidence supporting their efficacy in treating knee OA. Despite dietary recommendations published annually, there is still a need to debunk many myths that are not confirmed by current evidence. The significant research gaps require more extensive, controlled studies to establish evidence-based dietary recommendations (particularly carbohydrates, dietary fiber, and antioxidant intake). This comprehensive review provides insight into the various indications for the impact of nutrition on knee OA, focusing on key nutrients such as carbohydrates, fats, proteins, antioxidants, and selected micronutrients, providing the clinician with ready-to-implement nutritional modifications. Such an analysis may help clinicians and patients incorporate dietary strategies into treating knee OA, emphasizing the need for personalized, sustainable approaches.

## 1. Introduction

Osteoarthritis, the most prevalent degenerative joint disease, represents a significant health challenge, affecting millions worldwide [1]. With an aging global population, over 130 million individuals will be affected by OA by 2050 [2]. Current therapeutic approaches focus primarily on pain management and functional improvement. Recently, as recommended by the World Health Organization, a wealth of data has highlighted the growing potential of dietary interventions as an adjunct to conventional therapies to alleviate symptoms associated with OA [3]. For example, omega-3 fatty acids, particularly those found in fish oil, have been shown to exert anti-inflammatory effects and reduce cartilage degradation in OA [4,5]. Similarly, polyphenols have demonstrated potential in reducing oxidative stress, joint pain, and inflammation [6]. Emerging evidence also highlights the benefits of high-protein diets in preserving muscle mass and supporting joint function in OA patients [7]. Additionally, diets rich in anti-inflammatory components, such as vitamins C and D, curcumin, and flavonoids, have beneficial effects in preclinical and some clinical settings [8,9]. The role of popular supplements like glucosamine and chondroitin remains contentious; while some studies report symptomatic relief, others suggest minimal or no benefit [10].

In this paper, we aim to provide a comprehensive review of the role of diet in OA, analyzing both the evidence-based benefits and the potential risks. The key dietary components, such as anti-inflammatory nutrients, high-protein diets, and antioxidants, are examined. The facts discussed and myths debunked paint a background, although evidence is still lacking in some areas. By addressing the strengths and limitations of available research, this review offers a balanced perspective, guiding clinicians and patients toward informed dietary choices in OA management. The innovative approach of broadly discussing current guidelines for lifestyle and OA therapy, distinguishing common myths, and highlighting the facts is a strength of this review. The recommendations presented in a form ready for implementation in clinical practice will facilitate clinicians and dietitians in establishing individually tailored dietary recommendations in accordance with personalized medicine.

## 2. Materials and Methods

### 2.1. Search Strategy

**Figure 1:**
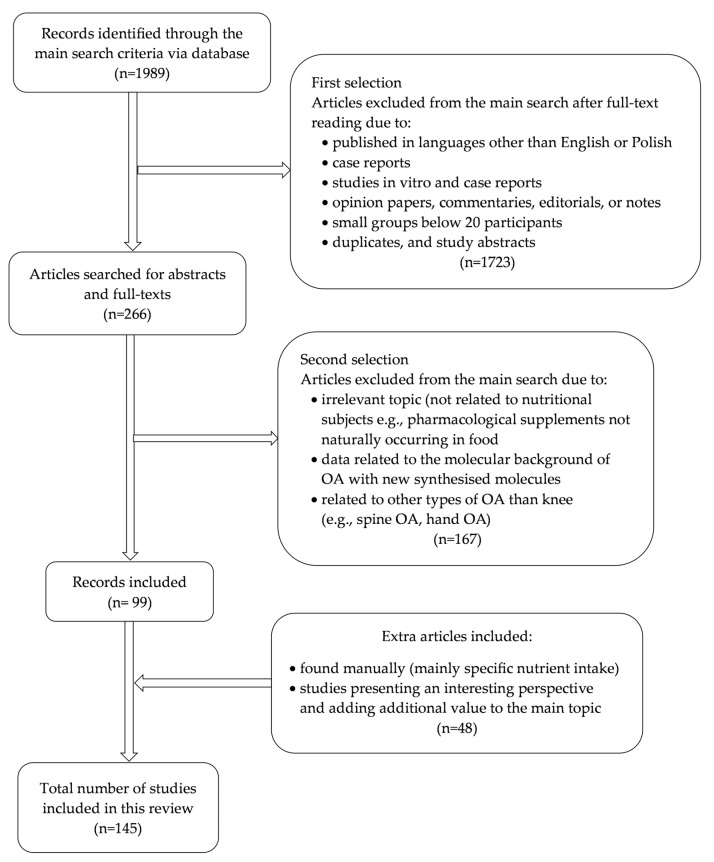
Diagram of the search strategy.

The initial analysis of extracted data from Pubmed and Web of Science (Embase) articles consisted of the title of the study, year of publication, the aims and objectives of the study, the study type/design (e.g., experimental studies, meta-analyses, case reports, commentaries, etc.), sample size, the age range of the study group, nutrient intake in traditional or interventional diets, the specific nutrients or pharmaceutical supplement interventions, and the main findings of the studies (Figure 1). Two independent reviewers reviewed each record cited in this review; no automation tools were used in this process.

PubMed and Web of Science (Embase) were used to search for literature published in English from January 2000 to December 2024 to identify relevant real-world publications on the influence of diet on knee osteoarthritis. Most of the articles were found using combinations of Boolean operators “AND/OR”, search terms, and synonyms for the keywords. The text words contained in the titles and abstracts of relevant articles and the MeSH terms used to describe the articles were used to develop a complete search strategy for PubMed. During the research, the keywords used to search articles were “knee osteoarthritis”, OR “diet”, “nutrients”, OR “knee osteoarthritis”, and “protein”, “fat”, “fatty acids”, “carbohydrates”, “dietary fiber”, “antioxidants”, “vitamin D”, “SYSADOA or glucosamine or chondroitin or avocado-soybean unsaponifiable” and “prevention and control”. There were no limitations on race, age, gender, or ethnicity. Related articles linked to key publications were searched for. Original research, review articles, and original research that reports on community-based or hospital research studies about OA in the population were selected. The remaining articles were identified manually by searching the reference lists of the original studies included in the review after searching two databases. The incidence rate (the number of new cases per population in a given period) of clinical manifestations of OA and its treatment complications was taken from reference articles.

### 2.2. Study Selection

After removing duplicates, the titles and abstracts of retrieved articles were screened. Full texts of retrieved publications were reviewed and marked for inclusion if they (1) included knee osteoarthritis patients who used specific dietary components, (2) were conducted in clinical practice, or included animal studies (data using animal models were only cited to explain the disease background or in case of lack human data), and (3) presented real-world evidence. Papers were excluded if they did not fit into the study’s conceptual framework.

### 2.3. Criteria for Inclusion

The review included studies conducted in OA patients without restrictions based on race, gender, age, study type/design, or setting. The review considers mainly studies analyzing various nutrient influences on OA conditions. Articles that investigated nutrient intake and its impact on animal cartilage and joint functions were also included, particularly in the case of a lack of specific data on humans. The review encompassed experimental study designs and observational studies (including prospective and retrospective cohort studies). Case–control studies and analytical cross-sectional studies were also included. To precisely analyze the connection between diet and OA risk, the study included only data describing the quality and quantity of nutrient intake.

### 2.4. Criteria for Exclusion

Excluded studies comprised articles published in languages other than Polish and English, duplicates, and study abstracts. In vitro studies and case reports were also excluded. Studies that involved analysis of nutritional products (molecules) that do not occur naturally in the diet were excluded. The review did not include opinion papers, commentaries, editorials, or notes.

Our preliminary search yielded 1989 articles. The detailed manual analysis also included articles describing specific nutrient intake or analysis/scoping review in the context of knee osteoarthritis (*n* = 47). After assessing articles for eligibility, 146 were finally included in the final analysis. This process is visualized in Figure 1.

Duplicates and study abstracts. In vitro studies and case reports were also excluded. Studies that involved analysis of nutritional products (molecules) that do not occur naturally in the diet were excluded.

## 3. Prevalence

Knee OA is a prevalent musculoskeletal disease worldwide, and its prevalence increases with age. Epidemiological data indicate that the prevalence of knee OA increased from 2.0% in 1996 to 3.6% in 2015 [2]. Among the elderly, its prevalence is exceptionally high, affecting approximately 15.0% of individuals aged over 85 [11]. Moreover, knee OA has been identified as the seventh leading cause of years lived with disability (YLD) in adults aged 70 years and older, with an age-standardized prevalence exceeding 5.5% worldwide in 2020 [12].

Interestingly, the review by Safiri et al. highlights a significant increase in the prevalence of moderate to severe knee OA from 3.7% in 1990 to 26.7% in 2017 (17-year follow-up period) [13]. These data underscore knee OA’s growing public health burden and the need to develop effective strategies for this disease.

## 4. The Impact of Nutrition on OA

Clinical studies have shown much evidence that diet can potentially affect OA [14,15,16,17,18,19,20,21,22]. Obesity is a major risk factor for OA associated with damage to cartilage tissue caused by excess body weight [16]. The study by Raud et al. showed that among 391 patients with knee OA, obesity of class I occurred in 57.0%, and obesity of class II/III in 14.6% of patients [14]. Many clinical trials have shown a positive relationship between the occurrence of knee osteoarthritis and metabolic syndrome, suggesting some involvement of metabolic factors in the development and progression of this disease (besides inflammatory changes and mechanical damage) [15,16,17,19]. Moreover, in the course of osteoarthritis, regardless of the presence of metabolic syndrome, a higher risk of cardiovascular diseases is observed [20,21,23]. This creates the need to implement appropriate prevention, including lifestyle modification and dietary behavior changes. Thus, adequately planned nutrition is integral to treating and preventing excessive body weight, metabolic disorders, and osteoarthritic changes, particularly in the knees and hips.

Conversely, weight loss reduces pain and improves function in knee osteoarthritis (meta-analysis). The study by Messier et al. points out that weight loss over 18 months achieved by a combination of diet and exercise is the most effective (meta-analysis). Such a regimen caused the most significant weight loss, efficiently diminished pain, and improved joint function compared to patients using only dietary restrictions or exercises [24]. Therefore, an adequate nutritional regimen combined with specific exercises that increase muscle strength seems to be the most effective and beneficial in managing knee OA.

### 4.1. Protein

A Western-type diet is an example of a dietary pattern associated with increased inflammation markers such as IL-6 and *C*-reactive protein. It is rich in red, processed meat, high-fat dairy products, and refined grains [25,26,27]. Moreover, it contributes to increased white adipose tissue, which can cause excessive weight-bearing joint and cartilage load, leading to joint surface degradation [28]. A high-protein diet may induce hyperuricemia and increase the risk of chronic diseases like gout, hypertension, kidney disease, and cardiometabolic disorders [29].

Protein is a crucial macronutrient in the diet, and its deficiency causes loss of muscle mass and strength [30]. Therefore, optimal protein intake is essential to maintain proper physical ability. The recommended protein intake for healthy adults aged 18 years and over is 0.8 g/kg/day [31]. Because older adults have been considered at high risk of insufficient dietary protein intake, especially those with chronic conditions like OA or sarcopenia, the protein intake requirements should be higher than 1.2–1.5 g/kg/day [32,33]. Conversely, a high-protein diet has been shown to have higher inflammatory potential and increase the risk of knee OA [34].

Recent studies have provided new insights into the metabolomics of OA and identified new metabolic markers of OA involving amino acid metabolism. Zhai et al. demonstrated that the branched-chain amino acid (BCAA)-to-histidine ratio was associated with knee OA, highlighting its potential as an osteoarthritis biomarker [35]. BCAAs are not produced by the body and must be obtained from food to ensure proper tissue growth [36]. BCAAs (valine, leucine, and isoleucine) are essential amino acids that play a role in protein synthesis and energy metabolism [37]. Increased plasma BCAA levels from supplements may be related to increased inflammation due to the production of pro-inflammatory cytokines such as IL-1 and IL-2, tumor necrosis factor-alpha (TNFα), and interferon-gamma (INFɣ), and lead to the destruction of articular cartilage [35,38]. Furthermore, several studies have also shown a positive association between plasma BCAA concentrations and the risk of metabolic conditions, including cardiovascular diseases, type 2 diabetes, and insulin resistance, which are associated with the metabolic background of OA [18,39,40,41].

A study by Zhang et al. demonstrated that knee OA patients have decreased plasma concentrations of arginine compared to healthy controls. The authors of this study suggest that the depletion of arginine in OA could be understood as an effort to repair the damaged cartilage by producing more ornithine, proline, and polyamines [42]. Further longitudinal human studies are needed to confirm the role of BCAAs and arginine metabolic pathways in OA progression and investigate the effect of their dietary intake on joint function [36,38].

### 4.2. Fat Intake in OA

**Figure 2:**
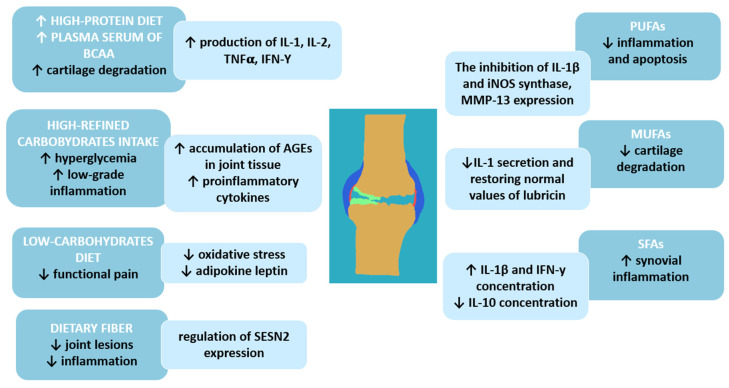
Macronutrients and osteoarthritis—molecular background; BCAAs—branched-chain amino acids; IL-1—interleukin-1; IL-2—interleukin-2; IL-10—interleukin 10; TNF-α—tumor necrosis factor; IFN-γ—interferon-gamma; iNOS—inducible nitric oxide synthase; MMP-13—matrix metallopeptidase 13; AGEs—Advanced glycation end products; SESN2—sestrin-2 protein; PUFAs—polyunsaturated fatty acids; MUFAs—monounsaturated fatty acids; SFAs—saturated fatty acids; ↑—increase; ↓—decrease.

Fat is another component of daily food rations, which has an essential effect on joint health. A high-fat diet is related to excessive weight and increased joint loading [43]. Nevertheless, dietary guidelines recommend that total fat intake be between 20 and 35% of total energy delivered daily and that consumption of saturated fatty acids be lower than 10% of total energy intake daily [44,45]. This suggests that not only quantity but also the quality of fat is crucial in improving the condition of the cartilage (Figure 2). Furthermore, a higher percentage of energy intake from fat is associated with a higher risk of developing OA. Interestingly, there was no similar observation between the percentage of energy intake from carbohydrates or protein and the risk of OA [46].

The study by Lu et al. demonstrates that increased total dietary fat or saturated fatty acid (SFA) intake is associated with increased OA progression [43]. A high amount of SFAs in the diet may also increase synovial inflammation in patients with OA, suggesting a link between disease development and progression and dietary fat composition [46]. In a study by Sekar et al. [47] conducted in an animal model, a diet rich in SFAs increases levels of pro-inflammatory cytokines—interleukin-1 beta (IL-1β) and IFN-γ—and decreases levels of anti-inflammatory interleukin 10 (IL-10).

A primary food source of MUFAs is olive oil, which also has a beneficial effect on OA. An animal model by Musumeci et al. shows that intake of olive oil and physical activity are associated with lower articular cartilage degradation by restoring the expression of lubricin to typical values and lowering IL-1 secretion after anterior cruciate ligament transection in mice [48]. There is a significant association between a high intake of MUFAs (31.9 g/day) and polyunsaturated fatty acids (PUFAs, about 17.1 g/day within the Q4 quartile) and the reduced risk of radiographic progression of OA [43].

Among fatty acids, PUFAs are the most researched regarding the risk and progression of articular cartilage due to their anti-inflammatory properties. Consistent evidence suggests that PUFAs are mediators and regulators of inflammation by reducing the expression of inflammatory markers involved in the pathogenesis of cartilage degeneration, such as IL-1β and inducible nitric oxide synthase. Furthermore, EPA acts as a precursor for eicosanoids, which reduce apoptosis induced by oxidative stress in OA chondrocytes by inhibiting metalloproteinase 13 (MMP13). MMP-13 (collagenase 3) is the key enzyme in the cleavage of type 2 collagen and plays a pivotal role in the breakdown of cartilage in osteoarthritic joints [49,50].

The recommended intake for total PUFAs (including both EPA (eicosapentaenoic acid) and DHA (docosahexaenoic acid)) is established at 250 mg daily as an adequate intake (AI) standard for adults [44]. A meta-analysis by Deng et al., including only randomized controlled trials with double-blind studies, shows that supplementation of *n*-3 PUFAs relieves pain and improves joint function in patients with OA. However, additional supplementation of omega-3 fatty acids for people who meet adequate dietary intake does not increase the effect [49]. Similarly, the study by Hill et al. shows that there are no greater benefits of supplementation with high doses of fish oil (4.5 g) than low doses (0.45 g) in 202 patients with knee OA after two years of observation (randomized, double-blind trial). The authors did not find a difference between groups in cartilage volume loss. However, this study had no placebo group for ethical reasons [51]. Therefore, additional supplementation of PUFAs should be considered in patients with knee OA, particularly those on a vegan diet. Moreover, some findings demonstrated a positive correlation between fish oil intake and cardiometabolic risk, which is relevant for patients with OA [52,53]. The main nutritional sources of EPA and DHA are fish oils. Therefore, a beneficial diet pattern for OA patients may be a Mediterranean diet because of the high consumption of fish and olive oil. Mediterranean habits are also well known for their positive effects on cardiovascular and metabolic diseases, and such a diet is particularly beneficial for obese osteoarthritic individuals [54,55,56].

### 4.3. Carbohydrates

**Figure 3:**
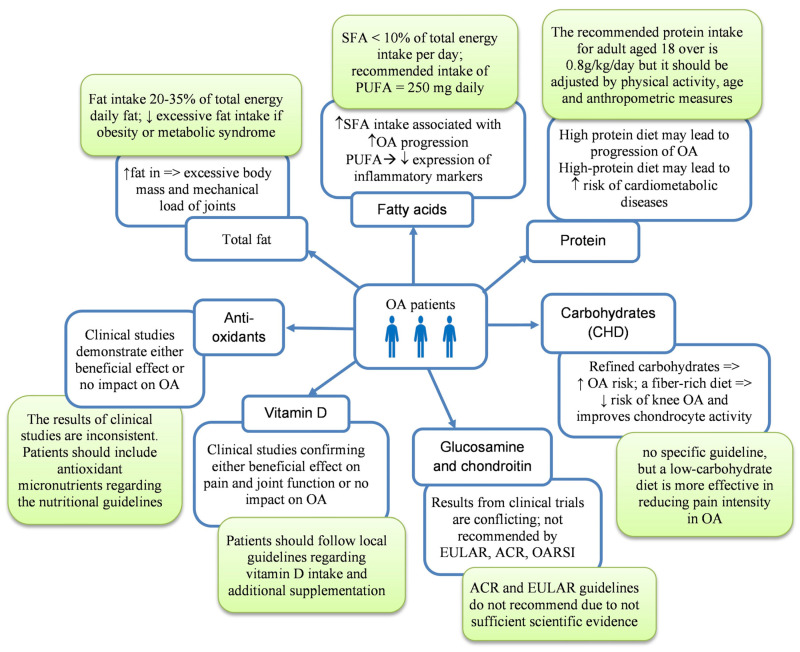
Influence of selective nutrients on OA patients with current recommendations of their intake (green boxes); ↑—increase; ↓—decrease. OA—osteoarthritis; OARSI—Osteoarthritis Research Society International; SFAs—saturated fatty acids; PUFAs—polyunsaturated fatty acids; ACR—American College of Rheumatology; EULAR—European Alliance of Associations for Rheumatology.

Diets with varying carbohydrate content have been studied for their effects on knee osteoarthritis (Figure 3). The largest body of research on the impact of these nutrients on OA comes from data describing obese individuals who consume a diet high in carbohydrates and simple sugars [57]. Increased body weight places mechanical stress on weight-bearing joints such as the knee and hip, accelerating cartilage degeneration and increasing joint pain [58].

Excess carbohydrate consumption is strongly linked to weight gain and systemic inflammation, which can worsen OA symptoms [57]. Recent data show that high-carbohydrate diets (≥50% of daily energy intake), particularly those rich in refined carbohydrates, are associated with increased OA risk due to their role in promoting inflammation and weight gain [59]. Animal studies also suggest that specific carbohydrate compositions (e.g., refined versus complex carbohydrates) may influence OA progression, with refined carbohydrates exacerbating joint damage even if the diet does not contain high amounts of fat [57,59]. Also, a diet high in refined carbohydrates may lead to the development of type 2 diabetes [60]. Unfortunately, chronic hyperglycemia stimulates low-grade inflammation and oxidative stress by producing pro-inflammatory cytokines and increasing the accumulation of advanced glycation end products (AGEs) in joint tissues [17,61]. In clinical studies, a highly refined carbohydrate diet can amplify systemic inflammation, a key factor in OA progression, thereby worsening pain and stiffness in affected joints [62].

In contrast, a low-carbohydrate diet (≤30% of daily energy intake) is more effective in reducing pain intensity in OA patients compared to a low-fat diet, suggesting that a reduction in carbohydrate intake may alleviate OA symptoms by reducing oxidative stress and adipokine leptin [58]. Such a diet may help individuals with OA manage their body weight effectively and reduce systemic inflammation, potentially improving joint health and relieving arthritis symptoms [59]. The sources of healthy carbohydrates, such as fruits, vegetables, and whole grains, provide nutritional benefits without triggering inflammation, while refined sugars and processed foods should be avoided [62]. These dietary adjustments can also mitigate comorbidities, such as cardiovascular diseases, prevalent in individuals with OA [28].

Dietary fiber plays a significant role in managing osteoarthritis (OA) and may lower the risk of developing this condition. Research indicates that a fiber-rich diet is associated with a reduced risk of knee OA. A meta-analysis examining two long-term studies found that participants with higher fiber intake had a significantly lower risk of developing symptomatic OA. Specifically, in 869 knees with symptomatic OA and 152 knees with radiographic OA from the large cohorts (Osteoarthritis Initiative, *n* = 4796), the dietary analysis of dietary total fiber in specific quartiles showed that fiber was inversely associated with the risk of symptomatic OA (*p* trend < 0.03) with a significantly lower risk in the highest versus lowest quartile [63]. Fiber-rich foods, such as fruits, vegetables, whole grains, and legumes, not only provide essential nutrients but also help reduce systemic inflammation, which is a critical factor in OA progression [64]. The anti-inflammatory effects of dietary fiber may be attributed to its influence on gut microbiota composition, promoting beneficial bacteria that help maintain joint health. It was proved that Bacillota-dominant gut microbiota supports joint health by maintaining chondrocyte activity and promoting increased expression of Sestrin-2 protein in joint tissue. Moreover, increased fiber intake (a diet comprising 20% plant polysaccharides and a substantial presence of microbial carbohydrates, including corn, soy, wheat, and oats) has been linked to improved chondrocyte activity and reduced joint inflammation, which can delay the onset of OA [65]. Incorporating adequate dietary fiber into one’s diet can thus provide multiple benefits for individuals with OA, including better weight management and reduced inflammation, ultimately leading to improved joint health and decreased symptoms [28].

Patients with knee OA should consult healthcare professionals or nutritionists to develop a personalized dietary plan. This ensures that their carbohydrate intake supports symptom management and overall health, tailored to their unique needs and associated diseases.

### 4.4. Antioxidants

Recently, most attention has been paid to the role of antioxidants in OA, as oxidative stress might be involved in developing this disease. Several plausible mechanisms of antioxidants’ influence on OA include restoring the antioxidative capacity of chondrocytes and suppressing inflammation by inhibiting pro-inflammatory signaling pathways. Oxidative stress caused by an imbalance between the production of reactive oxygen species (ROS) and their clearance by the antioxidant defense system induces senescence in chondrocytes by inhibiting the synthesis of extracellular matrix and a decrease in the synthesis of proteoglycan and type 2 collagen (Col2a1) [66,67,68,69,70].

Although the role of oxidative stress in the development of OA is undeniable, many clinical studies demonstrate conflicting results on the effects of antioxidants on OA risk and progression. For example, several studies report the beneficial influences of vitamins E and C on cartilage conditions [66,71,72], while others have not confirmed these findings [73,74,75,76,77]. The conflicting results can be related to the methods used, the specificity of the study group, the number of patients, the traditional diet, the type of OA, and the observation period. Nevertheless, most of the studies highlight the beneficial effect of vitamin E. For instance, a large cohort study (*n* = 29,406) by Veen et al. has shown a positive correlation between a high intake of dietary vitamin E and risk of OA (such an association was not observed in the case of vitamin C, beta-carotene, and non-enzymatic antioxidant capacity). However, the food intake was assessed only once at the beginning of the study, and the dietary changes that affected the results were not monitored [66]. The beneficial effect of vitamin E was also proved in seventy-two patients with severe knee OA, who were randomly assigned to the study group receiving a 400 IU vitamin E supplement or a placebo. Plasma and synovial fluid oxidative markers were significantly lower in participants receiving vitamin E. Thus, vitamin E can act as a disease-modifying agent for OA due to its high bioavailability and anti-inflammatory properties [78].

Another dietary antioxidant in the diet is selenium. This microelement is crucial in supporting antioxidant defense systems, participates in the metabolism of thyroid hormones, improves brain function, and controls reproductive functions [79,80]. The natural sources of selenium are mainly products of animal origin (red meat, processed meat, fish, milk, and cheese). If they are consumed in higher-than-recommended amounts, it may lead to a higher risk of the development of metabolic disorders, obesity, and inflammation [81,82]. Unfortunately, low selenium intake is observed in the elderly population, who have a higher risk of developing OA [79,83]. In a study by Qu et al., serum selenium levels were inversely associated with the risk of OA (*n* = 361,141 individuals, data from UK Biobank) [84]. However, such a relation was not confirmed by the large cross-sectional analysis of Deng and Tan (*n* = 26,620), which showed high dietary selenium intake might be associated with the risk of OA, pointing that dietary selenium intake below 100 μg does not increase the risk of OA (analysis based on data from NHANES cohort—National Health and Nutrition Examination Survey; 2003–2016) [85].

Zinc is another nutrient characterized by antioxidative properties, which play a key role in bone development and growth by activating proteins in their homeostasis [86]. Moreover, zinc can reduce oxidative stress markers by inhibiting the production of *C*-reactive protein and blocking the adhesion of molecules on macrophages and monocytes, protecting the body against inflammatory processes. Zinc is mainly found in meat, seafood, and nuts [86,87]. Zheng et al. [87] have confirmed a positive association between elevated zinc intake and a slowdown in the progression of subchondral sclerosis in OA patients, underlining the crucial protective role of zinc in bone health. However, some studies suggest that zinc potentially negatively affects OA by negatively impacting cartilage integrity [88,89].

Further studies are needed to confirm the antioxidative properties of various nutrients and the role of different nutritional and environmental factors in the development of OA. Nevertheless, patients should include antioxidants in their diet regarding good health policy as they are also sources of other beneficial nutrients such as vitamins, minerals, dietary fiber, and phytocompounds [8,25].

### 4.5. Glucosamine and Chondroitin

Glucosamine is an endogenous amino-monosaccharide synthesized from glucose and a precursor in synthesizing glycosylated proteins and lipids (Table 1). Its highest concentration is in articular cartilage. Chondroitin is a component of the extracellular matrix of articular cartilage, which has a significant role in preserving osmotic pressure [90,91]. Glucosamine and chondroitin are known as symptomatic slow-acting drugs (SYSADOAs), which can potentially improve symptoms of OA with better tolerability and decreased risk of adverse events, which often occur while treatment with non-steroidal anti-inflammatory drugs (NSAIDs). They may be combined with pharmaceutical-grade or dietary food supplements, which are not tested under strict quality standards of pharmaceuticals and prompt concern regarding safety and efficiency of usage [92,93]. Unfortunately, they are not as effective as was suspected. A meta-analysis by Zhu et al. showed that combination therapy of chondroitin and glucosamine has no significant effect on pain and stiffness reduction and better joint function, compared with a placebo group in knee and/or hip OA (glucosamine improved stiffness compared to the placebo, but the differences were not statistically significant). There was also no significant difference in the incidence of adverse events. However, oral chondroitin in monotherapy was more effective than placebo in relieving pain (*p* = 0.003) and improving physical function (*p* = 0.002) [94]. Similar findings were described by Simental-Mendía et al. [90]. Thus, oral glucosamine or chondroitin sulfate administration alleviates pain in knee OA patients; however, combining the two supplements does not provide any added benefit.

Another SYSADOA is avocado–soybean unsaponifiable (ASU), a natural plant extract from unsaponifiable fractions of avocado and soybean extracts. The main components of ASU are phytosterols, beta-sitosterol, canola stanols, and soya stanols, fat-soluble vitamins that are rapidly incorporated into cells [95,96]. Clinical studies demonstrate the “chondroprotective” potential of ASU by preventing cartilage degradation due to inhibiting the release and activity of matrix metalloproteinases (MMP-2, 3, 13) and increasing tissue inhibitors of these catabolic enzymes (TIMP-1) [97,98]. The efficacy and safety of ASU in patients with hip or knee osteoarthritis (OA) were evaluated in a systematic review and meta-analysis by Simental-Mendía et al. [99]. The study showed a positive effect only in symptomatic knee OA, but with no significant alterations in cartilage structure. The findings also confirmed that ASU is well tolerated in doses of 300–600 mg/d without significant adverse events compared with a placebo.

The number of studies assessing the combination of glucosamine and chondroitin therapy is limited. Therefore, the Osteoarthritis Research Society International does not recommend using chondroitin and glucosamine (including formulations with pharmaceutical grade) (OARSI 2019). Similarly, EULAR and ACR do not recommend SYSADOAs (including chondroitin sulfate, glucosamine, diacerein, and ASU) because their effectiveness has not been proven (except for a slight improvement in osteoarthritis of the hands in the case of chondroitin sulfate [100,101,102,103].

Conversely, the European Society for Clinical and Economic Aspects of Osteoporosis, Osteoarthritis and Musculoskeletal Diseases (ESCEO, 2019) [104] recommendations for the management of knee osteoarthritis strongly recommend the use of SYSDOAs, including pharmaceutical-grade crystalline glucosamine sulfate and chondroitin sulfate. Neither guidelines recommend the treatment of glucosamine hydrochloride, glucosamine sulfate, and/or chondroitin sulfate if they are non-pharmaceutical grade [104,105,106].

**Table 1:** The role of symptomatic slow-acting drugs in osteoarthritis.

	SYSADOA in Osteoarthritis		
	Chondroitin	Glucosamine	ASU
Pharmaceutical forms	Sulfate or hydrochloride	Glucosamine sulfate, glucosamine hydrochloride, and *N*-acetyl glucosamine	Extracts from fruits and seeds of avocado and soybean oil (mainly in a 1:2 ratio but also 1:1, 1:3, and 2:1) [107]
Doses	400–1500 mg [94]	1500 mg/d [102,108,109]	300–600 mg [110]
Stiffness	Meta-analysis (*n* = 4079 patients, 13 trials): 800–1200 mg chondroitin vs. placebo does not improve stiffness; *p* = 0.604 [94]	1500 mg/day improved stiffness, but the effect was insufficient (*n* = 2859) [109] Meta-analysis (*n* = 4079 patients, 13 trials): 1500 mg glucosamine vs. placebo improved stiffness (*p* = 0.048) [94]	↓ Lequesne index, but no significant difference between 300 mg/d and 600 mg/d of ASU (*n* = 260; *p* < 0.01) [110] Meta-analysis (*n* = 664 OA patients; 4 RCTs) 300 mg/d of ASU vs. placebo resulted in ↓ Lequesne index (*p* = 0.0003) [111]
Pain	1200 mg of chondroitin vs. placebo—positive effect on pain (*n* = 3082 patients; 12 trials; *p* = 0.003) [94] 1200 mg chondroitin (*n* = 318) vs. placebo (*n* = 314) had no significant effect on pain (*p* = 0.17) [108]	Glucosamine showed no significant effect pain compared vs. placebo (*n* = 2845; 14 trials; *p* = 0.170) [94] 1500 mg/d glucosamine (*n* = 317) vs. placebo (*n* = 313)—no significant effect (*p* = 0.30) [108]	↓ Pain (no significant difference between 300 mg/d and 600 mg/d doses, *n* = 260; *p* < 0.01) [110] 300 mg/d of ASU vs. placebo ↓ pain reduction (*p* = 0.04; meta-analysis, *n* = 664 OA patients, 4 RCTs) [111]
Physical function	Improved function vs. placebo (*p* = 0.02) [94]	No significant effect vs. placebo (*p* = 0.073) [94]	Improved the functional ability (300 mg/d dose, *n* = 164) [112]
Tolerance	Good safety profile [113]	Low toxicity [114]	Well tolerated in doses 300–600 mg/d [99,112]
Side effects	Well tolerated—no significant difference vs. placebo (*n* = 7172 in 14 trials, meta-analysis) [94]	No major adverse events ↑ glucose serum [114] Epigastric pain, tenderness, heartburn, diarrhea, nausea [97]	300 mg/d or 600 mg/d glucosamine (*n* = 260) vs. placebo—mild adverse events—mainly gastrointestinal [110].
EULAR recommendations	Conditional recommendation in hand OA (EULAR 2008) [100]	No recommendation [100]	No evidence for clinical efficiency (EULAR 2008) [100]
ACR recommendations	Strongly recommended against in patients with knee and/or hip OA, conditionally recommended for patients with hand OA [114]	Strongly recommended against in patients with knee, hip, and/or hand OA [114]	No information
NICE recommendations (2022)	No information	Not recommended [115]	No information
OARSI recommendations (2019)	Not recommended [106]	Not recommended [106]	Not recommended [106]

↓—decrease; ↑—increased; *n*—number of patients; ASU—avocado–soybean unsaponifiable extracts; Lequesne index—presence of pain, discomfort, morning stiffness; OA—osteoarthritis; RCTs—randomized controlled trials.

### 4.6. Vitamins in Osteoarthritis

Vitamin D is crucial for bone health. Because serum vitamin D concentrations in patients depend on sun exposure, recommendations of the amount of vitamin D needed from the diet and additional supplementation are different between geographical locations. The European Food Safety Authority (EFSA) set the adequate intake of vitamin D for adults at 15 mg/day to achieve a serum 25(OH)D concentration near or above the target of 50 nmol/L [116]. Natural food sources of vitamin D are fish, egg yolks, milk, and dairy products [117,118]. It is also known that the capacity of human skin to produce vitamin D decreases in old age, which is one of the risk factors for OA [119,120].

Vitamin D increases bone mass, prevents loss, and maintains muscle function. However, the role of vitamin D in OA is controversial [121]. Some clinical studies have shown that vitamin D supplementation has a beneficial effect on pain and joint function [122,123,124], while others have indicated no significant influence on these parameters in patients with OA. A systematic review and meta-analysis by Zhao et al. demonstrated that vitamin D supplementation (ranging from 800 to 6000 UI) improves joint function and pain (measured by WOMAC, Western Ontario, and McMaster University (Universities Osteoarthritic Index)) in knee OA patients. However, no strong evidence was found for preventing structural progression in knee OA patients [122].

The Framingham study shows that low dietary intake and serum vitamin D levels are associated with an increased risk of knee OA [125]. It was later confirmed by Bergink et al., but only in subjects with low lumbar spine body mass density [126]. Conversely, the prospective study by Konstari et al. showed that low serum 25(OH)D3 levels were not associated with an increased risk of developing either hip or knee OA over 22 years [127]. Independent of these results, early screening for vitamin D deficiency in patients with decreased bone density is important.

The Guidelines for Preventing and Treating Vitamin D Deficiency (2023) by Płudowski et al. [118] recommend adjusting doses of cholecalciferol (or calcifediol) to serum 25(OH)D concentration, age, body weight, sun exposure of the patient, dietary habits, and lifestyle in the general population. Overweight, obesity, and metabolic syndrome, which are often associated with OA [16,19,128], belong to the risk groups of vitamin D deficiency and require special attention to their evaluation based on regular serum 25(OH)D tests [118].

Management and treatment guidelines issued by ACR and OARSI (both in 2019) conditionally recommend against the use of vitamin D due to limited and questionable health benefits [103]. The EULAR and ACR recommendations did not comment on vitamin D use in osteoarthritis [100,114].

Since clinical studies researching the role of vitamin D in the risk and progression of OA are inconsistent, OA patients should be regularly screened for possible vitamin D deficiency and follow local guidelines and recommendations regarding intake and supplementation of vitamin D [118,129,130].

## 5. Diet and Lifestyle Prevention of Knee Osteoarthritis

The questionnaire survey, including patients with OA and rheumatoid arthritis (RA), showed that nearly 75% of OA patients declared that their dietary habits affected their health. Still, only 4% received professional dietary counseling after diagnosis of the disease [131]. This fact is related to the lack of trained staff educating OA patients on appropriate diets. In addition, a lot of misinformation (especially on social media) related to the appropriate diet gives rise to many myths about the diet in this disease, which have no scientific justification (Table 2).

The EULAR recommendations for the nonpharmacological management of hip and knee OA include education, physical therapy, and maintaining a healthy weight, as overweight and obesity are substantial risk factors for OA, particularly knee OA [132]. Recent studies have also provided new insights into the importance of metabolic factors in the progression of osteoarthritis changes, which increases understanding of maintaining a healthy and nourishing diet as part of treatment. The previously mentioned Mediterranean diet may be a beneficial dietary pattern for OA patients due to its anti-inflammatory properties and as a source of omega-3 fatty acids, polyphenols, and antioxidants. However, limited evidence supports that thesis [133,134]. Further large-scale studies are needed to investigate the impact of diet on OA.

A limitation of this review is the lack of clinical trials analyzing the selected dietary components (only studies on animal models are available), which limits the possibility of nutritional recommendations to mere suggestions. Due to the lack of sufficient clinical data to determine the amount of the intervening substance in the diet (e.g., the amount of fiber consumed), it is difficult to determine the beneficial amount of a specific nutrient in the OA diet. Given the small patient groups in some cited studies, it is problematic to extrapolate these data to a larger population [43]. Unfortunately, not all studies include the homogenous OA group (e.g., advanced OA vs. newly diagnosed OA); therefore, specific nutrients’ efficacy may be overestimated or underestimated (e.g., some nutrients may be adequate in early OA but do not affect advanced disease). Therefore, further randomized controlled trials are needed to determine the amount of specific nutrients and their impact on different stages of OA in large populations.

The strength of this review is the discussion of numerous nutrients in the context of current knowledge and recently published guidelines, both nutritional and rheumatological, issued by scientific institutions. Sixteen current recommendations and guidelines of scientific organizations and societies are compared with observational studies. In addition to the intake of basic macro- and micronutrients, we also discuss the effect of SYSADOA on OA, citing available data and official opinions on their effectiveness. The review also includes practical aspects describing the amount of each nutrient in a diet that protects against OA, which may be helpful in the daily practice of general practitioners, family doctors, dietitians, rheumatologists, and patients themselves. The gathered myths and the compiled facts allow for an easy composition of a well-balanced diet for patients with OA of the knee.

**Table 2:** Myths and facts about nutrients’ influence on osteoarthritis development.

Analyzed Component	Nutritional Components in Osteoarthritis	
	Myths	Facts
Energy intake	Energy intake has no impact on the progression of OA. Physical activity is an effective method to prevent OA in obese patients.	Excessive calorie intake leads to the development of obesity, which increases joint and cartilage load and contributes to cartilage degradation [8,24,135,136]. Long-term weight loss (a few years) caused by a combination of diet and exercise is the most effective method to efficiently diminish pain and improve joint function compared to patients using only dietary restrictions or only exercises [24].
Protein	A high-protein diet has a protective effect against OA and prevents OA development.	Protein should be consumed as recommended, adjusted by sex, age, anthropometric parameters, and physical activity. The recommended protein intake for healthy adults aged 18 and over is 0.8 g/kg/day [31]. A high-protein diet may lead to higher inflammatory potential [26,27]. The most important is the quality of the dietary protein, particularly the amount of BCAAs (valine, leucine, and isoleucine), which increase the pro-inflammatory state (synthesis of IL1, IL-2, TNFα, INF-ɣ, and OA development) [35,37,38]. Conversely, a low arginine concentration can be caused by it being used for repairing damaged cartilage by producing more ornithine, proline, and polyamines [42].
Total fat	Fat intake has no impact on the progression of OA.	High-fat diets have been shown to exacerbate the progression of OA, and increased SFA intake is associated with OA progression [43]. Recommended intakes: total fat 20–35% of total calories per day, SFA < 10% of total calories per day [31,137].
Fatty acids	High doses of *n*-3 PUFAs decrease the risk of OA.	An adequate dose of EPA + DHA at 250 mg daily for adults relieves pain and improves joint function in patients with OA [46,51,52,54,138]; however, there are no more significant benefits of supplementation with high doses of fish oil (4.5 g) than low doses (0.45 g) [51]. Recommendations of daily consumption of PUFAs (EPA + DHA) are 250 mg daily [44,139].
Carbohydrates	Increased carbohydrate intake does not affect the progression of OA	A low-carbohydrate diet may benefit individuals with knee OA [58]. Dietary fiber may lower the risk of OA and reduce inflammation.
Antioxidants	All dietary antioxidants have a beneficial influence on OA development.	Recent data deliver evidence of the beneficial effect of vitamin E [69,81] and selenium [79,83,84]. The data on other antioxidants (e.g., zinc, vitamin C) are inconclusive [66,71,72,73,74,75,76,77,87,88,89].
Vitamin E and vitamin C	Vitamins E and C lower the risk of OA and improve its symptoms	The results of clinical studies are inconsistent. However, a diet with recommended vitamin E and C intake maintains good health, and products containing these vitamins are rich in minerals, dietary fiber, and phytocompounds [8,66]. Moreover, the recommended vitamin C intake varies from 40 to 110 mg/day, and vitamin E is 12 mg/day to 19 mg daily for adults, depending on the country [140,141].
Selenium	Higher selenium intake is beneficial due to its antioxidant capacity	Selenium is a crucial antioxidant, but its higher intake can lead to selenosis and an increased risk of obesity and metabolic disorders [79,81]. The RDA for selenium is 55 µg (0.7 µmol)/day (men and women) [142].
Zinc	Higher intake of zinc improves joint functions by reducing inflammation	Some studies confirm the anti-inflammatory effect of zinc on the cartilage. On the other hand, some findings suggest zinc hurts joints [87,88,89]. EFSA’s estimated average requirements are from 6.2 to 10.2 mg/day for women and 7.5 to 12.7 mg per day for men [143].
Glucosamine and chondroitin	Background glucosamine and chondroitin treatment have better outcomes than treatments with NSAIDs	Despite the good safety profile of glucosamine and chondroitin, there are not enough RCTs that would confirm better outcomes in OA [106,114]. Moreover, EULAR and ACR guidance strongly recommend against the use of these supplements in knee OA [100,114].
Vitamin D intake	Vitamin D improves pain and joint function in OA	There is a lack of strong evidence on the role of vitamin D in OA prevention. Patients should follow local guidelines regarding vitamin D intake and additional supplementation [122,129]. The EFSA recommends an intake of vitamin D for adults at 15 mg/day to achieve a serum 25(OH)D concentration near or above the target of 50 nmol/L [116].

Abbreviations: BCAAs—branched-chain amino acids; EFSA—European Food Safety Authority; EPA—eicosapentaenoic acid; DHA—docosahexaenoic acid; OA—osteoarthritis; RDA—Recommended Dietary Allowance; RCTs—randomized controlled trials.

## 6. Conclusions

The relationship between dietary patterns and joint health is gaining increasing recognition. With the increasing prevalence of obesity and its consequences for joint function, it is imperative to address diet as part of the treatment of OA, including dietary evidence-based recommendations. The quality of protein, fat, and carbohydrates is important in the nutrition of patients with OA. A diet high in saturated fats, refined carbohydrates, and high-protein animal products (red, processed meats, high-fat dairy products) can lead to excessive cartilage loading and the development of metabolic disorders that will affect the progression of OA and reduce the patient’s quality of life. Fat sources rich in MUFAs and PUFAs, as well as foods rich in dietary fiber, may benefit joint health and cardiometabolism. The evidence regarding the effect of antioxidants on OA is limited due to several limitations of methodological background in current studies (small number of patients, lack of exact dose of nutrient intake in experiments). The role of vitamin D in OA is inconsistent. Patients should include trace minerals and vitamins with antioxidant properties in their diet as a good health policy recommended by guidelines. It is necessary to educate patients by qualified medical professionals who consistently dispel common myths and provide solid knowledge based on evidence-based medicine and nutrition.

Further high-quality studies are needed to confirm the clinical efficacy of SYSADOA. Therefore, most guidelines do not recommend its use due to limited evidence. Future large-scale studies should be planned to better understand the relationship between nutrients and the development of knee OA. In addition, randomized controlled trials may allow for the estimation of not only the amounts of nutrients but also the interactions between them and the precise effects on patients with different stages of OA.

## Abbreviations

The following abbreviations are used in this manuscript:

↑	increased
↓	reduced
ACR	American College of Rheumatology
AGEs	advanced glycation end products
AI	adequate intake
ASU	avocado–soybean unsaponifiable extracts
BCAAs	branched-chain amino acids
DHA	docosahexaenoic acid
EFSA	European Food Safety Authority
EPA	eicosapentaenoic acid
ESCEO	European Society for Clinical and Economic Aspects of Osteoporosis, Osteoarthritis and Musculoskeletal Diseases
EULAR	European Alliance of Associations for Rheumatology
IL-1	interleukin 1
IL-10	interleukin 10
IL-1β	interleukin-1 beta
IL-2	interleukin 2
INFɣ	interferon-gamma
Lequesne index	presence of pain, discomfort, morning stiffness
MMP	matrix metalloproteinases
MUFAs	monounsaturated fatty acids
N	number of patients
NSAIDs	non-steroidal anti-inflammatory drugs
OA	osteoarthritis
OARSI	Osteoarthritis Research Society International
PUFAs	polyunsaturated fatty acids
RCTs	randomized controlled trials
RDA	Recommended Dietary Allowance
ROS	reactive oxygen species
SFAs	saturated fatty acids
ss	statistically significant
SYSADOAs	symptomatic slow-acting drugs
TNFα	tumor necrosis factor-alpha
WOMAC	Western Ontario, and McMaster University Universities Osteoarthritic Index
YLD	years lived with disability
